# The longitudinal association between lifetime mental disorders and first onset or recurrent suicide ideation

**DOI:** 10.1186/s12888-019-2328-8

**Published:** 2019-11-06

**Authors:** Derek de Beurs, Margreet ten Have, Pim Cuijpers, Ron de Graaf

**Affiliations:** 1 Netherlands Institute of Health Services Research, Utrecht, Netherlands; 2 Department of Clinical, Neuro and Developmental Psychology, Vrije Universiteit Amsterdam, Amsterdam, the Netherlands; 3 Netherlands Institute of Mental Health and Addiction, Utrecht, The Netherlands

**Keywords:** Suicidal behavior, Comorbidity, Epidemiology

## Abstract

**Background:**

Although the cross-sectional association between mental disorders and suicide ideation is well studied, less is known about the prospective association. In this paper, we estimated among those without 12-month suicide ideation at baseline, the association between a wide variety of common mental disorders at baseline and suicide ideation within the 6-year follow-up period, after controlling for history of other mental disorders and demographic variables.

**Methods:**

Data were used from the Netherlands Mental Health Survey and Incidence Study-2 (NEMESIS-2), a prospective representative adult cohort study with baseline (*n* = 6646) with a 6-year follow-up period. Lifetime mental disorders were assessed at baseline with the Composite International Diagnostic Interview 3.0. Within the longitudinal design, participants with first time or recurrent suicide ideation were defined follows: having no suicide ideation in the 12 months before the baseline assessment, and reporting to have had seriously thought about suicide between baseline and the 6-year follow-up period. Multiple logistical regression was used to estimate the longitudinal association between suicide ideation and a specific mental disorder while controlling for comorbidity and baseline variables. To account for the prevalence of a disorder in the population, for each disorder, the population attributable risk proportion (PARP) was calculated.

**Results:**

2.9% (*n* = 132) of the participants that did not report suicide ideation in the past 12 months at baseline reported suicide ideation at follow-up. Of these 132 cases, 81 (61%) experienced suicide ideation for the first time in their lives and could be viewed as first onset cases. 51 (39%) reported recurrent suicide ideation. After controlling for comorbidity, the only two disorders that were significantly related to suicide ideation at follow-up were lifetime major depressive disorder (MDD) and generalized anxiety disorder (GAD). PARP for MDD was 47.8 and 16.6% for GAD.

**Conclusions:**

After controlling for all other mental disorders, a lifetime history of MDD and GAD were related to suicide ideation at follow-up. For clinical practice, this indicates that patients with a history of MDD or GAD stay vulnerable for suicide ideation, even though they did not report suicide ideation in the past year.

## Background

Suicide is a major global public health problem, with an estimated 800,000 suicides per year [1]. Although difficult to assess, it its estimated that for every person who died by suicide, there are around 20 people who attempted suicide. Suicide ideation are even more common. In different studies, the 12 months prevalence of suicide ideation was estimated to be around 3%, indicating that globally, each year millions of people consider life not worth living for at least a period of two weeks (e.g. Kessler et al., [2]; Ten Have et al., [3]). The association between a history of mental disorders and suicide and suicidal behavior (i.e. suicide ideation and attempts) is well established (e.g. Bolton and Robinson, [4]; Harris and Barraclough, [5]; Nock et al., [6]). Indeed, one of the most well established statistics in suicide prevention is that mental disorders play an important role in 90% of all suicides [7, 8]. Although this number has been up for debate [9], many cross-national population based studies confirmed the association between a history of mental disorders and suicide ideation, suicide attempts and completed suicides [4, 10, 11]. In high-income countries, after controlling for comorbidity, lifetime mood disorders such as major depressive disorders (MDD) were more strongly related to suicidal behavior in comparison to other disorders such as anxiety of substance use [11]. Most population-based studies are based on cross-sectional data, and therefore provide no information on the development of suicidal behavior over time [4, 10–12]. Longitudinal studies are scarce, so we know relatively little about the sequential relationship between lifetime mental disorder and later onset or recurrence of suicidal behavior. Knowing which disorders are uniquely related to suicidal behavior over time is important for a better understanding, prediction and treatment of suicidal behavior [10]. Analysis of a community sample survey with a 13 year follow-up indicated that especially major depressive disorder (MDD) and substance abuse were related to the first onset of suicide ideation and suicide attempts, although the authors did not control for comorbidity [13]. A population based survey from the UK with an 18 month follow-up found that the presence of mental illness was the best predictor of the first onset of suicide ideation, although no formal diagnostic interviews were conducted [14]. A study using Dutch longitudinal population based data (NEMESIS-1), found that after adjustment for comorbidity, life events and vulnerability factors such as neuroticism, mood and anxiety disorders increased the odds of first onset suicide ideation and suicide attempts [3]. One hypothesis that aims to explain this prolonged vulnerability for suicide ideation after an initial episode of a psychiatric disorder comes from the differential activation model [15, 16]: during a psychiatric episode, an association is formed between sad mood and dysfunctional, negative thinking. After the initial episode, as soon as a recovered patient experiences any small change in mood, this almost automatically results in negative thinking and the activation of dysfunctional self-schema’s which increase the chance of suicide ideation. In the current paper, we used data of the second Dutch longitudinal population-based study (Netherlands Mental Health Survey and Incidence Study-2: NEMESIS-2), to estimate the longitudinal association of a wide variety of common mental disorders and suicide ideation after controlling for demographic variables and comorbidity.

Suicide and suicidal behavior are a complex phenomenon, and the end result of the interaction of psychiatric, psychological, biological and social factors [8, 17]. Information on the history of psychiatric disorders will therefore only provide a part of this complex picture. However, detailed information on for example key psychological constructs such as entrapment is often not available in a patient medical record, whereas history of psychiatric illness will be [18, 19]. Knowing which psychiatrist disorders are most strongly associated with suicide ideation over time will help professionals such as general practitioners to decide which patient should asked more in detail about suicide ideation and plans.

Given the evidence for the association of MDD and suicide ideation, and the high level of comorbidity of for example anxiety and personality disorders with MDD, we hypothesize that after controlling for other psychiatric disorders, MDD will have the strongest association with suicide ideation at follow-up.

## Methods

The data used in this study comes from a population based psychiatric epidemiologic survey called the Netherlands Mental Health Survey and Incidence Study-2 (NEMESIS-2: De Graaf et al. [20]). It replicates and updates the first Netherlands Mental Health Survey and Incidence Study (NEMESIS-1: Bijl et al. [21]) conducted from 1996 to 1999. NEMESIS-2 is based on a multi-stage, stratified random sampling procedure of households, with one respondent randomly selected in each household. Data collection of the first wave (T0) started in November 2007 and lasted until July 2009 and resulted in 6646 respondents providing written informed consent and completing the assessment. The interviews were done by 98 professional interviewers of field work agency GfK. Given the sensitive topic of the interviews, they had to be at least 25 years old. Most interviews were administrated at the home of the respondent. Medical ethical approval was obtained from the national Medical Ethics Review Committee for Institutions on Mental Health Care (METC 12–255). The respondents were found to be representative of the general population, with a slightly underrepresentation of younger respondents. Three years after a respondent finished the first wave, he/she was approached for the second wave (T1). A total of 5303 persons (80.4%) could be interviewed again. A similar procedure was followed for the third wave: 3 years after T1, all respondents were again approached, resulting in 4618 (87.8%) persons to be re-interviewed. Except for bipolar disorder, attrition between T0 and T2 was not significantly associated with individual 12-month mental disorders or with lifetime suicidal behavior at T0. More details on the design and method can be found in de Graaf et al. [20].

### Diagnoses of lifetime mental disorders

The presence of any lifetime common mental disorders at baseline was determined using the Composite International Diagnostic Interview 3.0 (CIDI 3.0). The CIDI is a fully structured diagnostic interview that can be administered by lay persons, and was developed and adapted for use in the World Mental Health Survey Initiative [22]. A validation study found the results of the CIDI 3.0 to be comparable with blinded clinical reappraisal interviews [23].

In this paper, we looked at the following disorders: mood disorders (i.e. major depression, dysthymia, and bipolar disorder), anxiety disorders (i.e. panic disorder, agoraphobia without panic disorder, social phobia, specific phobia, and generalized anxiety disorder) and substance use disorders (i.e. alcohol/drug abuse and dependence).

Borderline personality disorder (BPD) and antisocial personality disorders (APD) were measured at the second wave via questions from the International Personality Disorder Examination at the second wave [24]. For the 19.6% of participants that were missing at the second assessment (due to attrition), no borderline scores were available, and were given a score of 0 (no BPD).

### Suicidal behavior

Within the module on suicidal behavior of the CIDI 3.0, respondents were asked if they ever seriously thought about suicide and if they ever attempted suicide. These questions on lifetime history of suicide ideation and suicide attempt were listed in a booklet and the respondents were asked if any of the experience ever happened to them. The same questions were repeated at follow-up with the time frame limited to the period between assessments.

### Participants with first onset or recurrent suicide ideation

Participants with first onset or recurrent suicide ideation were defined follows: reporting no suicidal behavior in the 12 months before the baseline assessment, and reporting to have had seriously thought about suicide between baseline and the 6 year follow up period. People have first onset of suicide ideation at follow up when they have never in their life experienced suicide ideation before the baseline assessment, and then reported suicide ideation at follow up. When patients reported to have had thoughts about suicide in their life time (but not within the 12 months before baseline), and then (again) reported suicide ideation at follow up, we considered them to have recurrent suicide ideation.

### Statistical analysis

Data were weighted to ensure that the data were representative of the national population. The included demographic variables were: sex, job status and partner status (all dichotomous variables), age (continuous variable) and education (primary education, lower secondary education, higher secondary education, higher professional education/university).

We examined the cross-sectional relationships at baseline between lifetime mental disorders on the one hand and lifetime suicide ideation and suicide attempt on the other hand (Tables 1 and 2). Among the participants who at baseline reported no suicide ideation or suicide attempts in the 12 months before baseline, we examined the longitudinal relationships between lifetime mental disorders at baseline and subsequent first onset or recurrent suicide ideation within the six-year follow-up period (Table 3). Due to the low number of suicide attempts at follow-up, no separate analysis for suicide attempts was possible. For all tables, we conducted 4 sets of logistic regression analyses: unadjusted OR (OR-1), adjusted OR for demographic variables and other axis-I mental disorders except for those within its own main category (AOR-2), adjusted OR for demographic variables and all other axis-I mental disorders (AOR-3), and additionally for all other axis-II mental disorders (AOR-4).

### Population attribution proportion (PARP)

To estimate the proportion of cases of suicide ideation or suicide attempt that could be prevented if a specific disorder was successfully treated in all cases, one can calculate the population attribution proportion (PARP). PARP is calculated by subtracting the prevalence (or incidence or recurrence of suicidal behavior in the longitudinal analysis) in the group without a specific disorder from the prevalence (or incidence or recurrence) of suicidal behavior in the total sample. It shows how much of the suicidal behavior in the general population can be attributed to a specific disorder. We calculated the PARP for each lifetime mental disorder.

## Results

### Cross-sectional association between lifetime mental disorders and lifetime suicidal behavior

#### Lifetime history of suicide ideation

Table 1 shows that 83.0% of the respondents with a lifetime history of suicide ideation also reported a lifetime history of an axis-I disorder. More specifically, 65.2% had any lifetime mood disorder, 50.6% any anxiety disorder, and 38.0% any substance use disorder. 11.4% reported a history of antisocial or borderline personality disorder. Of the respondents with any axis-I disorder, 16.1% reported lifetime suicide ideation, compared to 25.4% of the respondents with any axis-II disorder. Univariate analysis showed that every assessed disorder was significantly related to suicide ideation. All odds decreased after controlling for demographic variables and comorbidity. Odds only decreased somewhat after adjusting for axis-II disorders (AOR-3 versus AOR-4). In the last analysis (AOR-4), all lifetime mood disorders, social phobia and generalized anxiety disorder (GAD), and all substance disorders except for alcohol dependence were significantly related to lifetime suicide ideation. Bipolar disorder had the highest odds ratio followed by major depressive disorder (MDD). However, as depression is far more prevalent in the population compared to bipolar disorder, PARP for MDD was 48.1, whereas the PARP for bipolar disorder was 5.9. In the univariate association, the odds for borderline personality disorder (BPD) were among the highest, but the odds decreased considerably when controlling for comorbidity, Also, due to the relative low prevalence, the PARP was only 3.9.

**Table 1:** Cross-sectional analyses of lifetime mental disorders and lifetime suicide ideation at baseline, in unweighted numbers, weighted column and row percentages and (un)adjusted odds ratios (OR) with 95% confidence intervals (CI)

	Total (*n* = 6646)	Suicide ideation (*n* = 566)		Suicide ideation				
	n	c%	r%	OR-1	AOR-2	AOR-3	AOR-4	PAF
Any mood disorder	1400	65.2	26.8	**9.73 [7.35,12.88]**	**6.34 [4.77,8.42]**	NA	NA	56.3
Major depression	1309	57.8	25.6	**7.64 [6.06,9.62]**	**5.30 [4.19,6.71]**	**5.90 [4.46,7.80]**	**5.83 [4.43,7.69]**	48.1
Dysthymia	91	8.2	50.7	**12.29 [7.46,20.25]**	**5.44 [2.87,10.32]**	**2.78 [1.54,5.00]**	**2.73 [1.48,5.04]**	6.9
Bipolar disorder	82	7.1	45.1	**9.69 [5.38,17.43]**	**2.39 [1.07,5.35]**	**7.17 [3.30,15.61]**	**6.88 [3.20,14.82]**	5.9
Any anxiety disorder	1335	50.6	21.4	**5.06 [4.05,6.32]**	**2.15 [1.65,2.78]**	NA	NA	38.5
Panic disorder	261	10.6	23.2	**3.61 [2.60,5.02]**	1.46 [0.97,2.18]	1.23 [0.79,1.91]	1.23 [0.78,1.95]	7.0
Agoraphobia	70	3.3	30.5	**4.97 [2.86,8.65]**	1.38 [0.73,2.62]	1.11 [0.56,2.20]	1.10 [0.55,2.20]	2.4
Social phobia	619	28.0	24.9	**4.71 [3.50,6.34]**	**1.76 [1.24,2.49]**	**1.56 [1.08,2.25]**	**1.56 [1.07,2.27]**	20.6
Specific phobia	552	18.4	19.4	**3.04 [2.22,4.16]**	**1.50 [1.01,2.24]**	1.29 [0.85,1.95]	1.26 [0.82,1.93]	11.5
Generalised anxiety disorder	304	16.5	30.4	**5.59 [4.07,7.68]**	**2.42 [1.62,3.61]**	**2.23 [1.49,3.34]**	**2.20 [1.46,3.31]**	12.5
Any substance use disorder	1128	38.0	16.5	**2.92 [2.30,3.70]**	**2.13 [1.65,2.74]**	NA	NA	23.4
Alcohol abuse	825	21.2	12.3	**1.69 [1.23,2.33]**	**1.57 [1.15,2.15]**	**1.48 [1.09,2.01]**	**1.45 [1.07,1.97]**	8.0
Alcohol dependence	113	8.4	34.4	**6.23 [3.97,9.79]**	**2.49 [1.29,4.79]**	**2.03 [1.05,3.91]**	1.86 [0.95,3.65]	6.5
Drug abuse	225	13.5	29.6	**5.21 [3.66,7.41]**	**3.21 [2.05,5.05]**	**3.07 [2.00,4.72]**	**3.05 [1.99,4.67]**	10.1
Drug dependence	107	9.1	33.8	**6.10 [3.65,10.21]**	**2.29 [1.11,4.70]**	**2.42 [1.19,4.92]**	**2.15 [1.01,4.58]**	7.0
Any axis-I mental disorder	2777	83.0	16.1	**7.60 [5.69,10.16]**	NA	NA	NA	70.3
Number of axis-I disorders								
0	3923	16.8	2.4	REF	NA	NA	NA	NA
1	1521	22.1	7.9	**3.53 [2.58, 4.83]**				
2	615	19.7	17.8	**8.87 [6.18, 12.72]**				
3 or more	587	41.4	37.1	**24.10 [16.47, 35.25]**				
Antisocial personality disorder	157	7.7	21.4	**3.18 [2.01,5.03]**	NA	**1.91 [1.08,3.37]**	**1.88 [1.05,3.35]**	4.9
Borderline personality disorder	58	4.7	46.6	**10.06 [5.37,18.84]**	NA	2.20 [0.56,8.58]	2.12 [0.56,7.98]	3.9
Any axis-2 mental disorder	206	11.4	25.4	**4.13 [2.83,6.01]**	NA	NA	NA	8.0

Bold: Significant OR at the 0.05 level, 2-sided test

*REF* Reference category

*NA* Not applicable

*OR-1* Unadjusted OR

*AOR-2* Adjusted for demographic variables (sex, age, education, living situation, working situation) and other axis-I mental disorders except for those within its own main category (e.g. *major* depression was adjusted for all anxiety and substance use disorders, whereas panic disorder was adjusted for all mood and substance use disorders)

*AOR-3* Adjusted for demographic variables (sex, age, education, living situation, working situation) and all other axis-I mental disorders (e.g. *major* depression was adjusted for dysthymia, bipolar disorder as well as all anxiety and substance use disorders, whereas panic disorder was adjusted for agoraphobia, social phobia, specific phobia, generalised anxiety disorder as well as all mood and substance use disorders)

*AOR-4* Adjusted for demographic variables (sex, age, education, living situation, working situation) and all other axis-I mental disorders (e.g. *major* depression was adjusted for dysthymia, bipolar disorder as well as all anxiety and substance use disorders, whereas panic disorder was adjusted for agoraphobia, social phobia, specific phobia, generalised anxiety disorder as well as all mood and substance use disorders) and all other axis-II mental disorders

*PAF* Population attributable fraction

#### Lifetime history of a suicide attempt

With regard to participants with a lifetime history of a suicide attempt, 89.2% of the participants also reported a lifetime history of an axis-I disorder, and 17.8% an axis-II disorder. When looking the other way around, 4.7% with a lifetime history of an axis-I disorder also had a history of suicide attempt, compared to 10.7% of those with an axis-II disorder. Univariate associations between disorders and suicide attempts were all significant, except for bipolar and alcohol dependence. After controlling for demographic variables and other axis-I and axis-II mental disorders (Table 2: see AOR-4), only MDD, bipolar disorder, GAD, and antisocial personality disorder (APD) were significantly associated with lifetime suicide attempt. MDD had both the highest odds after controlling for comorbidity and the highest PARP.

**Table 2:** Cross-sectional analyses of lifetime mental disorders and lifetime suicide attempt at baseline, in unweighted numbers, weighted column and row percentages and (un)adjusted odds ratios (OR) with 95% confidence intervals (CI)

	Total (*n* = 6646)	Suicide attempt (*n* = 149)		Suicide attempt				
	n	c%	r%	OR-1	AOR-2	AOR-3	AOR-4	PAF
Any mood disorder	1400	76.4	8.4	**13.86 [8.29,23.18]**	**8.76 [4.76,16.14]**	NA	NA	70.4
Major depression	1309	70.4	8.4	**11.13 [6.96,17.81]**	**7.11 [4.15,12.18]**	**8.24 [4.45,15.24]**	**8.16 [4.40,15.14]**	63.5
Dysthymia	91	13.4	22.3	**14.36 [6.11,33.74]**	**5.46 [1.93,15.44]**	**2.70 [1.04,6.98]**	2.59 [0.95,7.05]	12.2
Bipolar disorder	82	6.0	10.3	**5.28 [2.55,10.93]**	1.23 [0.41,3.64]	**6.64 [2.01,21.93]**	**6.22 [1.81,21.36]**	4.8
Any anxiety disorder	1335	58.6	6.6	**6.14 [4.01,9.39]**	**2.07 [1.24,3.45]**	NA	NA	48.5
Panic disorder	261	10.8	6.3	**3.21 [1.88,5.46]**	1.13 [0.63,2.03]	1.00 [0.52,1.94]	1.01 [0.51,2.00]	7.2
Agoraphobia	70	3.2	8.1	**3.94 [1.50,10.34]**	0.99 [0.33,2.96]	0.82 [0.25,2.68]	0.82 [0.26,2.60]	2.4
Social phobia	619	30.8	7.4	**4.62 [2.83,7.53]**	1.43 [0.80,2.56]	1.34 [0.71,2.54]	1.33 [0.69,2.57]	23.7
Specific phobia	552	21.0	5.9	**3.24 [1.97,5.31]**	1.23 [0.68,2.24]	1.13 [0.61,2.08]	1.08 [0.56,2.06]	14.2
Generalised anxiety disorder	304	20.7	10.3	**6.09 [3.52,10.55]**	**2.10 [1.23,3.58]**	**2.04 [1.18,3.53]**	**1.91 [1.11,3.28]**	17.0
Any substance use disorder	1128	44.1	5.2	**3.48 [2.21,5.47]**	**2.35 [1.41,3.93]**	NA	NA	30.9
Alcohol abuse	825	22.7	3.5	**1.79 [1.01,3.16]**	1.59 [0.87,2.90]	1.55 [0.87,2.77]	1.48 [0.80,2.73]	9.8
Alcohol dependence	113	8.8	9.7	**5.05 [2.53,10.07]**	1.74 [0.71,4.26]	1.75 [0.64,4.75]	1.64 [0.57,4.67]	6.9
Drug abuse	225	16.4	9.7	**5.42 [2.83,10.38]**	**2.43 [1.15,5.12]**	**2.27 [1.01,5.09]**	2.12 [0.94,4.78]	13.1
Drug dependence	107	8.5	8.5	**4.34 [1.64,11.45]**	1.28 [0.34,4.90]	1.35 [0.33,5.47]	1.00 [0.19,5.25]	6.4
Any axis-I mental disorder	2777	89.2	4.7	**11.57 [6.60,20.28]**	NA	NA	NA	81.1
Number of axis-I disorders								
0	3923	10.8	0.4	REF	NA	NA	NA	NA
1	1521	16.9	1.6	**4.01 [2.13, 7.55]**				
2	615	17.8	4.3	**10.94 [5.40, 22.15]**				
3 or more	587	54.4	13.1	**36.40 [19.59, 67.66]**				
Antisocial personality disorder	157	10.8	8.1	**4.20 [1.91,9.24]**	NA	**2.46 [1.06,5.72]**	**2.41 [1.04,5.59]**	8.1
Borderline personality disorder	58	8.5	22.9	**14.13 [7.01,28.47]**	NA	2.47 [0.89,6.87]	2.37 [0.86,6.51]	7.8
Any axis-2 mental disorder	206	17.8	10.7	**6.15 [3.36,11.27]**	NA	NA	NA	14.6

Bold: Significant OR at the 0.05 level, 2-sided test

*REF* Reference category

*NA* Not applicable

*OR-1* Unadjusted OR

*AOR-2* Adjusted for demographic variables (sex, age, education, living situation, working situation) and other axis-I mental disorders except for those within its own main category (e.g. *major* depression was adjusted for all anxiety and substance use disorders, whereas panic disorder was adjusted for all mood and substance use disorders)

*AOR-3* Adjusted for demographic variables (sex, age, education, living situation, working situation) and all other axis-I mental disorders (e.g. *major* depression was adjusted for dysthymia, bipolar disorder as well as all anxiety and substance use disorders, whereas panic disorder was adjusted for agoraphobia, social phobia, specific phobia, generalised anxiety disorder as well as all mood and substance use disorders)

*AOR-4* Adjusted for demographic variables (sex, age, education, living situation, working situation) and all other axis-I mental disorders (e.g. *major* depression was adjusted for dysthymia, bipolar disorder as well as all anxiety and substance use disorders, whereas panic disorder was adjusted for agoraphobia, social phobia, specific phobia, generalised anxiety disorder as well as all mood and substance use disorders) and all other axis-II mental disorders

*PAF* Population attributable fraction

### Longitudinal association between lifetime mental disorders and first onset or recurrence of suicide ideation

At follow-up, 2.9% (*n* = 132) of the participants at risk at baseline (i.e. those without 12-month suicide ideation at baseline; *n* = 4571) reported suicide ideation (Table 3). Of these 132 cases, 81 (61%) experienced suicide ideation for the first time in their lives and could be viewed as first onset cases and 51 (39%) reported recurrent suicide ideation. All univariate associations between baseline disorders and suicide ideation at follow up were significant, expect for bipolar disorder and alcohol abuse (Table 3: OR-1). After controlling for demographic variables and all other axis-I and axis-II disorders (Table 3: see AOR-4), only the associations between MDD and GAD and onset or recurrence of suicide ideation at follow-up remained significant. PARP for MDD was 47.8 and 16.6% for GAD.

**Table 3:** First onset or recurrence of suicide ideation in relation to baseline lifetime mental disorders among those at risk (*N* = 4571), in unweighted numbers, weighted percentages and (un)adjusted odds ratios (OR) with 95% confidence intervals (CI)

	No suicide ideation (*n* = 4439)	Suicide ideation (*n* = 132)	Suicide ideation				
	n (%)	n (%)	OR-1	AOR-2	AOR-3	AOR-4	PAF
Any mood disorder	847 (17.5)	73 (58.1)	**6.51 [3.92,10.81]**	**5.04 [2.72,9.34]**	NA	NA	48.2
Major depression	808 (16.6)	70 (57.2)	**6.70 [4.08,11.01]**	**5.44 [3.10,9.55]**	**4.94 [2.74,8.91]**	**4.86 [2.69,8.79]**	47.8
Dysthymia	44 (1.0)	11 (8.7)	**10.06 [3.51,28.84]**	**6.15 [1.68,22.43]**	3.00 [0.85,10.60]	2.49 [0.57,10.92]	7.6
Bipolar disorder	33 (0.8)	3 (0.9)	1.08 [0.32,3.64]	0.24 [0.05,contributed to1.24]	0.75 [0.10,5.63]	0.66 [0.09,4.82]	0.1
Any anxiety disorder	839 (18.8)	53 (47.8)	**3.90 [2.28,6.68]**	1.77 [0.85,3.67]	NA	NA	34.7
Panic disorder	169 (3.6)	11 (7.9)	**2.24 [1.02,4.93]**	0.95 [0.40,2.23]	0.91 [0.38,2.19]	0.94 [0.39,2.27]	4.2
Agoraphobia	37 (0.8)	7 (3.0)	**3.95 [1.58,9.91]**	1.53 [0.61,3.85]	1.23 [0.48,3.15]	1.35 [0.52,3.48]	2.1
Social phobia	387 (9.0)	28 (19.3)	**2.42 [1.32,4.43]**	0.98 [0.50,1.92]	0.94 [0.47,1.87]	1.00 [0.49,2.05]	11.0
Specific phobia	347 (7.7)	18 (15.9)	**2.22 [1.03,4.79]**	1.15 [0.42,3.15]	1.12 [0.39,3.19]	1.07 [0.35,3.24]	8.5
Generalised anxiety disorder	179 (4.0)	21 (20.4)	**6.18 [3.13,12.23]**	**2.68 [1.33,5.40]**	**2.66 [1.32,5.38]**	**2.44 [1.14,5.19]**	16.6
Any substance use disorder	711 (18.0)	44 (32.3)	**2.17 [1.26,3.74]**	1.49 [0.84,2.66]	NA	NA	16.9
Alcohol abuse	552 (14.1)	23 (17.5)	1.30 [0.68,2.49]	1.07 [0.57,2.01]	1.03 [0.54,1.94]	1.02 [0.52,1.98]	3.9
Alcohol dependence	58 (1.7)	8 (7.6)	**4.77 [1.60,14.21]**	2.59 [0.69,9.69]	1.92 [0.51,7.24]	1.96 [0.55,6.99]	5.8
Drug abuse	129 (3.4)	15 (16.0)	**5.26 [2.31,12.01]**	**2.72 [1.07,6.89]**	**2.61 [1.12,6.07]**	2.40 [0.96,6.04]	12.6
Drug dependence	47 (1.6)	10 (5.8)	**3.92 [1.49,10.30]**	1.43 [0.42,4.88]	1.56 [0.46,5.34]	1.37 [0.44,4.25]	4.2
Any axis-I mental disorder	1759 (40.2)	97 (81.2)	**6.38 [3.64,11.18]**	NA	NA	NA	67.7
Number of axis-I disorders							
0	2716 (61.0)	35 (18.8)	REF	NA	NA	NA	NA
1	1058 (23.6)	38 (31.4)	**4.27 [2.23,8.16]**				
2	393 (8.8)	24 (23.3)	**8.55 [4.19,17.46]**				
3 or more	272 (6.6)	35 (26.5)	**12.96 [6.47,25.98]**				
Antisocial personality disorder	91 (2.8)	12 (10.3)	**3.88 [1.65,9.13]**	NA	**2.36 [1.13,4.93]**	2.14 [0.99,4.63]	7.4
Borderline personality disorder	26 (0.6)	8 (8.0)	**13.76 [4.88,38.78]**	NA	**7.16 [1.04,49.45]**	6.68 [0.93,47.72]	7.2
Any axis-2 mental disorder	115 (3.4)	18 (16.9)	**5.66 [2.70,11.87]**	NA	NA	NA	13.5

Bold: Significant OR at the 0.05 level, 2-sided test

*NA* Not applicable

*OR-1* Adjusted for time between baseline and follow-up assessments

*AOR-2* Adjusted for time between baseline and follow-up assessments, demographic variables (sex, age, education, living situation, working situation) and other axis-I mental disorders except for those within its own main category (e.g. *major* depression was adjusted for all anxiety and substance use disorders, whereas panic disorder was adjusted for all mood and substance use disorders)

*AOR-3* Adjusted for time between baseline and follow-up assessments, demographic variables (sex, age, education, living situation, working situation) and all other axis-I mental disorders (e.g. *major* depression was adjusted for dysthymia, bipolar disorder as well as all anxiety and substance use disorders, whereas panic disorder was adjusted for agoraphobia, social phobia, specific phobia, generalised anxiety disorder as well as all mood and substance use disorders)

*AOR-4* Adjusted for time between baseline and follow-up assessments, demographic variables (sex, age, education, living situation, working situation) and all other axis-I mental disorders (e.g. *major* depression was adjusted for dysthymia, bipolar disorder as well as all anxiety and substance use disorders, whereas panic disorder was adjusted for agoraphobia, social phobia, specific phobia, generalised anxiety disorder as well as all mood and substance use disorders) and all other axis-II mental disorders

*PAF* Population attributable fraction

## Discussion

In this study, we investigated the associations of lifetime axis-I and axis-II disorders and suicide ideation over time using data from a large Dutch population based sample (NEMESIS-2). We hypothesized that after controlling for the presence of other mental disorders, MDD would have the strongest association with suicide ideation over time compared to other disorders.

Around 2.9% (*n* = 132) of the participants showed suicide ideation at follow up, which is comparable to results from the first NEMESIS study [25]. We found that most psychiatric disorders were significantly related to suicide ideation over time when we did not control for comorbidity. However, when controlling for comorbidity, the only two disorders that had a significant association with suicide ideation at follow up were lifetime MDD and GAD. This indicates that although it might seem that every psychiatric disorder is related to the risk for suicide ideation over time, the additional predictive power of most diagnosis above and beyond MDD or GAD is neglectable [8]. As hypothesized, the association of MDD with suicide ideation at follow-up was twice as strong as the association of GAD with suicide ideation. After controlling for other disorders, the odd ratios for dysthymia, borderline personality disorder and any anxiety disorders dropped most sharply, reflecting their high level of comorbidity with MDD. The odds for GAD also dropped but stayed significant, suggesting that GAD does predict suicide ideation above and beyond MDD even though GAD is highly related to MDD.

The finding that a lifetime history of MDD and GAD were related to the onset or recurrence of suicide ideation indicates that particularly these respondents have a long-time vulnerability for suicide ideation. As stated in the introduction, this is in line with the differential activation hypothesis, that states that patients become more vulnerable for negative ideation and suicide ideation after an initial psychiatric episode due to the strengthening of the association between sad mood and negative thinking [15, 16]. For clinical practice, this indicates that patients with a history of MDD or GAD stay vulnerable for suicide ideation, even though they report no suicide ideation in the past 12 months. This group is at higher risk to develop suicide ideation even after only a mild decrease in mood. Primary care physicians or other gatekeepers should therefore carefully monitor patients with a (history of) MDD or GAD especially after new life stressors such as a divorce, as they are more vulnerable to develop suicide ideation.

Given the high prevalence of MDD, we found a high PARP of 47.8%. This suggests that if we would be able to effectively treat all participants with MDD, and prevent the recurrence of MDD, a reduction of 47.8% of the onset or recurrence of suicide ideation can be expected. This is in line with the rationale of the perfect depression initiative of the Henry Ford Health System Perfect Depression Care Program. After they adopted a program that optimized their treatment of depression, suicidal behavior, including actual suicides decreased substantially [26]. Indeed, training of gatekeepers and general practitioners (GP) in the recognition of participants with a high risk for (recurrent) depression and suicide ideation are at the core of the multifaceted community prevention programs European Alliance Against depression [27]. The training of GPs and gatekeepers, in combination with a public awareness campaign and a program for high risk groups lead to a significant decrease in suicidal behavior in both Germany [27] and Hungary [28]. The program is currently being implemented in the Netherlands [29].

### Anxiety disorders

The unique role of anxiety disorders in the development of suicidal behavior remains unresolved, as prospective studies both confirmed and falsified the effect of anxiety disorders on suicide ideation [30–32]. In our findings, lifetime GAD at baseline was the only anxiety disorder with a unique association with lifetime suicide attempts at baseline and with the onset or recurrence of suicide ideation at 6-year follow-up. Future studies with for example a larger sample at baseline and a longer follow-up could clarify the role of (comorbid) anxiety in the development of suicidal behavior.

### Limitations

As stated in the introduction, suicide and suicidal behavior are the end result of the interaction of many different factors [8, 17]. In this study, we only used information on history of psychiatric illness to learn more about the unique contribution of psychiatric disorders. The last decade saw an interest in understanding the psychological process that underpin suicide ideation and the decision to act on suicide ideation [8]. Knowing the history of psychiatric illness is an important, but only small piece of the puzzle to predict suicide and suicidal behavior [33]. Still, given the limited availability of other pieces of information, understanding the longitudinal association of history of psychiatric illness with suicide ideation gives the professional something that can be of immediate use in very day clinical practice.

Another limitation is that in this study, we combined first onset and recurrent suicide ideation. One might argue that first onset and recurrent suicide ideation have partly different pathways. For example, the relationship between mental disorders and recurrent suicide ideation is mediated by previous suicide ideation, making our reported relation of mental disorders and recurrent suicide ideation partly spurious [34]. However, we did not have enough cases of first onset or recurrent suicide ideation to compare the separate effects, so we do not know how large the difference in association is. The main goal of this paper was however not to offer the best prediction of suicide ideation, but to present the longitudinal association of mental disorders and suicide ideation, and compare the association with other findings.

As with all survey-based studies, our study lacks validity due to the retrospective nature of the design. Even on a salient topic such as suicidal behavior, participants can answer inconsistently between assessments [35]. Still, reviews suggest that participants can recall experiences retrospectively with enough accuracy to provide valuable information [36]. Not all personality disorders were recorded in the data set. As controlling for BPD and APD did not affect the results much, we do not expect our associations to be affected by this omission. Also, in contrast to the presence of mood disorders, personality disorders were not assessed via a diagnostic instrument but by using a screener, which might limit the validity .

We were also not able to test the longitudinal association between lifetime mental disorders and suicide attempts due to the small number of suicide attempts at follow-up. Pooling of cross-national population-based surveys might provide us with enough cases to investigate this associations, at least when suicidal behavior has been reported using similar methods. Finally, our sample consisted of a general population sample. The longitudinal effects of a history of mental disorders will be likely to be different in a psychiatric population when compared to a population based sample. Indeed a history of psychiatric disorders may be less informative within a group that all have a history of psychiatric disorders. Several studies show that within a clinical sample, the longitudinal trajectories of risk factors such as hopelessness and emotional dysregulation may be best predictors of future suicide ideation and attempts [37, 38].

At baseline, 83% of participants with lifetime suicide ideation and 89.2% of participants with lifetime suicide attempt had any axis-I lifetime mental disorder. These results are in line with Sareen et al. [25], but higher when compared to findings from other studies [3, 10, 11]. In these studies, a mental disorder had to be reported before or in the year of onset of the suicidal behavior. In the current study, we did not focus only on prior mental disorders, but any lifetime mental disorders. Our results suggest that respondents can have had serious episodes of suicide ideation or behavior before they had a full-blown axis-I or axis-II disorder. Indeed, earlier analysis of the NEMESIS-2 data revealed that around 25% of the participants with onset suicide ideation and around 19% of the participants with onset suicide attempt had no prior mental disorder [3]. By focusing on prior mental health disorders only, other studies may have underestimated the role of suicidal symptoms in the development of axis-I or II disorders.

## Conclusion

After controlling for comorbidity, a lifetime history of MDD and GAD were related to suicide ideation at follow-up, indicating that these respondents have a long-time vulnerability for suicide ideation. For clinical practice, this indicates that patients with a history of depression or GAD stay vulnerable for suicide ideation, even though they do report suicide ideation in the past year.

## Data Availability

The datasets used and/or analysed during the current study are available from the corresponding author on reasonable request.

## Abbreviations

AOR: Adjusted odd ratio
APD: Antisocial personality disorders
BPD: Borderline personality disorder
CIDI 3.0.: Composite International Diagnostic Interview 3.0
GAD: Generalized anxiety disorder
MDD: Major depressive disorder
NEMESIS-2: The Netherlands Mental Health Survey and Incidence Study-2
OR: Odd ratio
PARP: Population attributable risk proportion
T0: Baseline assessment, first wave
T1: Follow-up assessment, second wave
T2: Follow-up assessment, third wave
